# CYP1B1 expression is induced by docetaxel: effect on cell viability and drug resistance

**DOI:** 10.1038/sj.bjc.6604195

**Published:** 2008-01-22

**Authors:** V G Martinez, R O'Connor, Y Liang, M Clynes

**Affiliations:** 1 National Institute for Cellular Biotechnology (NICB), Dublin City University, Glasnevin, Dublin 9, Ireland

**Keywords:** cytochromes *P*450, CYP1B1, drug resistance, breast

## Abstract

The cytochrome *P*450 CYP1B1 is consistently overexpressed in tumour cells as compared to their normal counterparts, but its precise role in drug resistance is yet to be defined. It has been reported that transfection of CYP1B1 results in increased resistance to docetaxel in V79 cells (McFadyen *et al*, 2001). In this study, we analysed changes in expression of CYP1B1 mRNA associated with pulse selection of MCF-7 cells with docetaxel. Docetaxel-selected MCF-7 cells (MCF-7 Txt), which showed increased resistance to this drug as compared to parental MCF-7 cells, showed a noteworthy increase in CYP1B1 mRNA expression, paralleled by increased ethoxyresorufin-*O*-deethylase (EROD) activity levels. This effect was not observed in cisplatin- or adriamycin-selected MCF-7 cells, or in docetaxel-selected colon, lung or pancreatic carcinoma cells. Short-term treatment with docetaxel induced CYP1B1 mRNA expression in MDA 453 and BT-20 breast carcinoma cells, but not in MCF-7 cells. Transfection of MCF-7 Txt cells with CYP1B1 siRNA did not significantly affect docetaxel-induced toxicity, but it decreased cell survival in the absence of drug. Preincubation of docetaxel with recombinant CYP1B1 did not affect drug toxicity in A549 cells. These results suggest that CYP1B1 does not directly inactivate docetaxel, but rather might promote cell survival in MCF-7 Txt cells, providing an explanation for its association with drug resistance.

Breast cancer affects a significant number of women worldwide and is a leading cause of cancer deaths (Chintamani *et al*, 2004). Current therapies for breast cancer include locoregional (surgical) treatment, anti-hormone therapy, radiotherapy and chemotherapy (O'Driscoll and Clynes, 2006). Chemotherapeutic treatment of breast cancer, although of great importance in the management of the disease, has limited benefits for many patients due to intrinsic or acquired resistance to the effects of anticancer drugs. Moreover, chemoresistance is often associated with a more aggressive phenotype with an increased tendency to invade and metastasise (Campbell *et al*, 2001).

Cytochromes *P*450 are a family of enzymes implicated in the biotransformation of both xenobiotics and endogenous compounds. Their primary functions are the synthesis of steroids and bile acids and the detoxification of many foreign substances, such as drugs and environmental agents.

CYP1B1 is the only member of the CYP1B subfamily; it catalyses the hydroxylation of 17-*β*-oestradiol at the C4 position (Jefcoate *et al*, 2000) and is involved in testosterone biotransformation as well (Shimada *et al*, 1999). It is also known to metabolise xenobiotics such as ethoxyresorufin, theophylline and caffeine (Shimada *et al*, 1997). CYP1B1 is constitutively expressed at the mRNA level in mammal steroidogenic tissues, such as rat granulosa cells (Dasmahapatra *et al*, 2002) and its expression can be induced in these and other tissues by peptide hormones, cAMP and ligands of the aryl hydrocarbon receptor (AhR). Cross-talk has been described between the oestrogen receptor (ER)*α* and the CYP1B1 expression pathways (Spink *et al*, 1998). Regulation of expression appears to be tissue-specific, since treatment with the AhR agonist 2,3,7,8-tetrachlorodibenzo-*p*-dioxin (TCDD) induces CYP1B1 mRNA in MCF-7 cells but not in HepG2 cells (Spink *et al*, 1994). More recently, regulation of CYP1B1 expression by microRNAs has been described (Tsuchiya *et al*, 2006).

Conflicting data exist in the literature regarding CYP1B1 expression. A number of studies failed to detect presence or activity of functional CYP1B1 protein in normal (nontumour) tissue (Murray *et al*, 1997; McFadyen *et al*, 1999); however, expression of CYP1B1 in normal tissue has been reported, although in much lower levels than in tumour tissue, at both mRNA and protein levels (Muskhelishvili *et al*, 2001; Gibson *et al*, 2003). In spite of these inconsistencies, most studies agree that CYP1B1 protein is commonly overexpressed in malignant as compared to normal tissue.

The significance of CYP1B1 overexpression in cancer is not yet fully understood, but the fact that it can inactivate flutamide, an antiandrogen used in the treatment of prostate cancer, with high efficiency suggests that this enzyme might play an important role in the development of resistance to some forms of chemotherapy (Rochat *et al*, 2001). Also, a significant decrease in the sensitivity of CYP1B1-transfected V79 Chinese hamster ovary cells to docetaxel as compared to the parental cell line has been described (McFadyen *et al*, 2001); cytotoxicity was restored by pretreating the cells with the CYP1 inhibitor *α*-naphtoflavone. Although transfection of CYP1B1 has been reported to confer resistance to docetaxel, no metabolites could be detected after incubation of this drug with recombinant CYP1B1 in a number of different conditions (Bournique and Lemarie, 2002). Thus, even though expression of CYP1B1 appears to be associated with drug resistance, its precise role remains obscure.

In this study, we report induction of CYP1B1 mRNA expression by docetaxel in breast cell lines, both after short-term treatment and pulse-selection. The docetaxel-mediated increase in CYP1B1 expression was cell type-specific, as lung, pancreas and colon cell lines pulse-selected with docetaxel did not show such induction. CYP1B1 small interfering RNA (siRNA) transfection resulted in decreased cell survival, but did not cause major changes in docetaxel toxicity. Overall, expression of CYP1B1 appears to promote cell survival in MCF-7 Txt cells; this effect might significantly contribute to the increase in drug resistance observed in these cells.

## MATERIALS AND METHODS

### Chemicals

Cell culture media were obtained from Gibco BRL (Paisley, UK); serum was from Sigma (Poole, UK). Docetaxel was obtained from Sanofi Aventis Pharmaceuticals (Surrey, UK), cisplatin was from Mayne Pharma Plc. (Warwickshire, UK) and paclitaxel was from Bristol-Myers Squibb (Dublin, Ireland). 5-fluorouracil was obtained from Faulding Pharmaceuticals (Warwickshire, UK) and adriamycin was from Ebewe (Unterach, Austria). Reverse transcription polymerase chain reaction (RT–PCR) reagents and all other chemicals were purchased from Sigma.

### Cell lines

MCF-7, BT-20, MDA 231 and MDA 453 breast carcinoma and A549 lung adenocarcinoma cells were obtained from the ATCC (American Type Culture Collection). BT-20 cells, MCF-7 cells and their pulse-selected variants were maintained in MEM supplemented with 10% fetal calf serum (FCS), 1 mM sodium pyruvate and 1 mM non-essential amino acids. MDA 231 and MDA 453 cells were maintained in RPMI supplemented with 10% FCS. A549 cells were maintained in a 1 : 1 mixture of DMEM and Ham's F12 medium supplemented with 5% FCS. Cell lines were cultured in a humidified atmosphere of 95% air/5% CO_2_. All docetaxel-selected variants were referred to as Txt, for example, MCF-7 Txt; adriamycin-selected variants were referred to as Adr and cisplatin as CisPt.

### Pulse selection of cells

MCF-7 cells were pulse-selected with adriamycin (4 pulses, once a week for 4 h, with 250 ng ml^−1^ adriamycin), cisplatin (9 pulses, once a week for 4 h, with 800, 100, 300, 300, 300, 300, 300, 350, 350 ng ml^−1^ cisplatin, respectively for each week) and docetaxel (6 pulses, once a week for 4 h, except for the first one, which was for a half hour, with 50, 10, 10, 13, 13, 13 ng ml^−1^ docetaxel, respectively) to generate the MCF-7 Adr, MCF-7 CisPt and MCF-7 Txt variants, correspondingly. Drug concentrations and pulse schedule were decided based on changes in cell morphology and resistance; a dynamic pulse schedule was used here according to our previous experience with this cell line.

### *In vitro* toxicity testing

Cytotoxicity testing of drugs was measured by the acid phosphatase assay as previously described (Martin and Clynes, 1991). Briefly, cells were seeded at 1 × 10^3^ cells per well in a 96-well plate and left to attach overnight in a 5% CO_2_ incubator at 37°C. The appropriate concentrations of drug were prepared freshly at 2 × their final concentration and added to the plate on the following day. The assay was terminated after a further 7-day incubation. The concentration of drug causing a 50% kill (IC_50_ of the drug) was determined using CalcuSyn (Biosoft, Cambridge, UK). Fold resistance was calculated as the ratio between IC_50_ values in parent and pulse-selected cells. All assays were performed at least in triplicate.

### RT–PCR

Adherent cells were grown in 75 cm^2^ flasks until approximately 80% confluent; mRNA was then extracted using TRI reagent (Sigma) according to the manufacturer's instructions. RNA was quantified spectrophotometrically at 260 and 280 nm using the Nanodrop (Nanodrop Technologies, Wilmington, DE, USA). One microgram of mRNA was then subjected to reverse transcription and the resulting cDNA amplified by PCR. The sequences of primers used for CYP1B1 amplification were GTATATTGTTGAAGAGACAG for the forward primer and AAAGAGGTACAACATCACCT for the reverse primer (Baron *et al*, 1998); *β*-actin was used as control. The mixture was amplified for 35 cycles using the following conditions: 94°C for 3 min, 49°C for 30 s, 72°C for 1 min. Polymerase chain reaction products were then separated on a 2% agarose gel and visualised by ethidium bromide staining on a UV transilluminator.

### Determination of EROD activity

Ethoxyresorufin-*O*-deethylase (EROD) activity of cells in culture was determined as previously described (Bandiera *et al*, 2005). Briefly, cells were incubated with a 400 nM solution of 7-ethoxyresorufin in PBS pH 7.4 at 37°C/5% CO_2_ for 60 min. After incubation, 200 *μ*l of the cell supernatant was transferred to an opaque 96-well plate and fluorescence were determined at 530 nm excitation and 590 nm emission in a fluorescence plate reader (Synergy HT; Bio-Tek, Winooski, Vermont, USA). The sample measurements were compared to a resorufin (Sigma; R3257) standard curve. Ethoxyresorufin-*O*-deethylase activity was then calculated as the measured amount of resorufin (in picomoles), divided by the total protein content of the sample (in milligrams) and by the total reaction time (in minutes).

### RNA interference

RNA interference analysis was performed using siRNAs chemically synthesised and purchased from Ambion Inc., Warrington, UK). Three different siRNAs for CYP1B1 were used (Ambion Ids. 2253, 105952 and 112548). Small interfering RNAs were introduced into the cells via reverse transfection with the transfecting agent siPORT™ *Neo*FX™ (Ambion Inc.). Conditions for transfection were optimised using kinesin siRNA (Ambion Inc.). Solutions of negative control (scrambled), kinesin and CYP1B1 siRNAs at a final concentration of 30 nM were prepared in optiMEM (Gibco™). NeoFX solutions were also prepared in optiMEM and incubated at room temperature for 10 min. After incubation, either scrambled, kinesin or CYP1B1 siRNA solution was added to each NeoFX concentration; these solutions were mixed thoroughly and incubated for a further 10 min at room temperature. Replicates of 10 *μ*l of the siRNA/NeoFX solutions were added to a 96-well plate. A total of 7.5 × 10^3^ cells in 100 *μ*l were then added to each well; the plates were mixed gently and incubated at 37°C for 24 h. After this period, the transfection mixture was removed from the cells and the plates were fed with fresh medium. Forty-eight hours after transfection, concentrations of the chemotherapeutic agent (at 2 × the final concentration) were added to the plates in replicates of four. The plates were assayed for changes in proliferation after a further 72 h using the acid phosphatase assay.

### Drug metabolism by microsomes

Drug metabolism was investigated in the microsomal fraction of recombinant insect cells (microsomes) expressing CYP1B1 and cytochrome *P*450 NADPH reductase (P450R) (Beckton Dickinson, Oxford, UK) or P450R alone. Fifty microlitres of a stock solution of the appropriate drug were prewarmed at 37°C in a total incubation mixture volume of 250 *μ*l containing potassium phosphate buffer (PBS, pH 7.4), MgCl_2_ (25 mM, 25 *μ*l) and NADPH (6.5 mM, 75 *μ*l). The reaction was initiated by the addition of 100 *μ*l of a 1 mg ml^−1^ solution of ice-cold microsomes to the incubation mixture, as recommended by the manufacturer. After an incubation period of either 30 or 60 min, the mixture was filter sterilised and used for toxicity assays as described above.

### Statistical analysis

Differences between cell lines were assessed using a two-tailed Student's *t*-test. A *P*-value <0.05 was deemed significant.

## RESULTS

### Expression of CYP1B1 in MCF-7 cells and their drug-resistant variants

CYP1B1 expression has been shown to be abundant in hormone-dependent tissues such as uterus, ovary and breast (Tsuchiya *et al*, 2005). A breast cell line, MCF-7, was therefore chosen as a model in which CYP1B1 expression might be studied.

MCF-7 cells were pulse-selected either with adriamycin, cisplatin or docetaxel to generate the MCF-7 Adr, MCF-7 CisPt or MCF-7 Txt variants, respectively. Toxicity assays were carried out in these cells to establish their resistance profile (Table 1). MCF-7 Adr were 4.8-fold more resistant to adriamycin than parent MCF-7 cells; similarly, MCF-7 CisPt were 2.1-fold more resistant to cisplatin, and MCF-7 Txt displayed a 2.8-fold increase in resistance against docetaxel as compared to MCF-7 cells. Some cross-resistance occurred in MCF-7 Adr and MCF-7 CisPt cells with docetaxel, but it was not observed with other drugs or in any of the other cell lines. Interestingly, MCF-7 Adr cells were more sensitive to cisplatin than parental cells.

**Figure 1:**
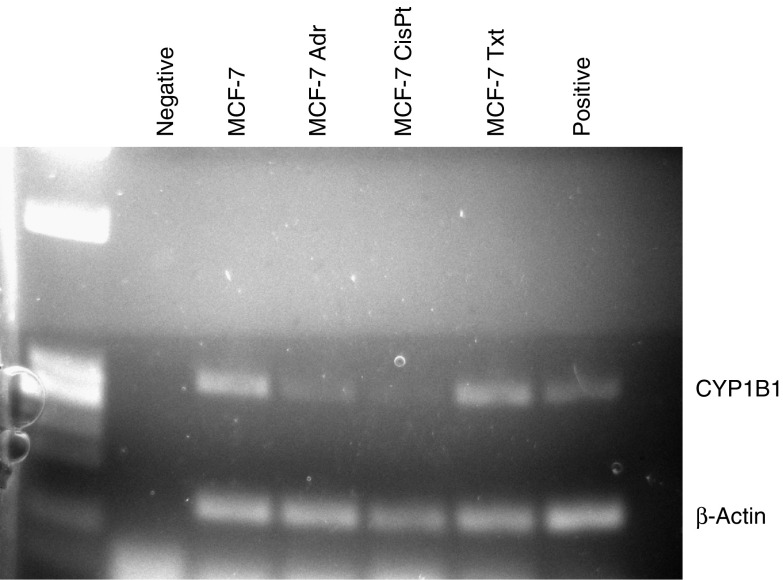
Expression of CYP1B1mRNA in MCF-7 cells and their resistant variants by RT–PCR. HL60 cells treated with TCDD were used as positive control. Ultrahigh purity (UHP) water was used as negative control.

Expression of CYP1B1 mRNA was measured in MCF-7 cells and their resistant variants by RT–PCR (Figure 1). The mRNA levels of this enzyme were increased in MCF-7 Txt cells as compared to parental cells. This upregulation of CYP1B1 expression was not observed in MCF-7 Adr or MCF-7 CisPt cells; furthermore, CYP1B1 mRNA levels appeared to be lower in these cells as compared to parental MCF-7 cells. To confirm that the increase in CYP1B1 transcription resulted in enhanced enzymatic activity, EROD activity was measured in MCF-7 and their docetaxel-selected variants. Activity was low but detectable in MCF-7 Txt cells, while no measurable activity could be detected in MCF-7 cells (Table 2).

### Expression of CYP1B1 in a panel of docetaxel-selected cells of various origins

**Figure 2:**
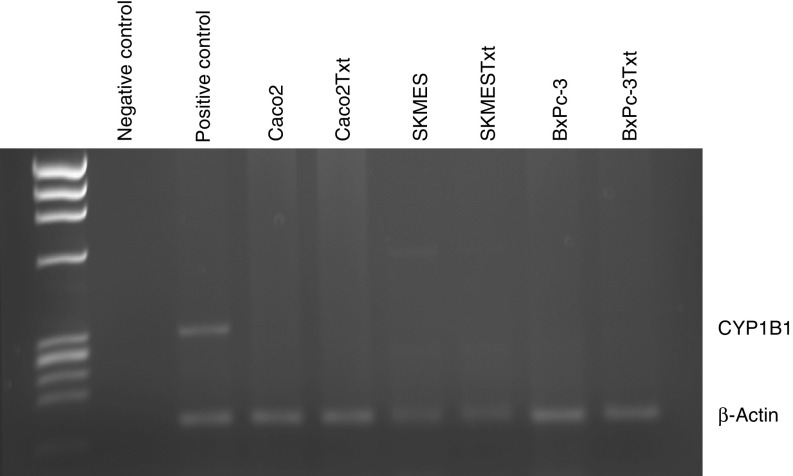
Expression of CYP1B1 mRNA in a panel of cell lines pulse selected with docetaxel and their respective parent cells by RT–PCR. HL60 cells treated with TCDD were used as positive control. Ultrahigh purity (UHP) water was used as negative control.

To find out whether increased expression of CYP1B1 was a common effect of docetaxel pulse selection, a panel of cell lines – colon carcinoma Caco2, squamous cell lung carcinoma SK-MES-1 and pancreatic carcinoma BxPc-3 cell – and their docetaxel-selected variants were analysed for CYP1B1 mRNA expression (Figure 2). No CYP1B1 mRNA was detected in any of these cell lines, whether pulse-selected or parent cells.

### Effect of docetaxel on CYP1B1 expression in a panel of breast cell lines

**Figure 3:**
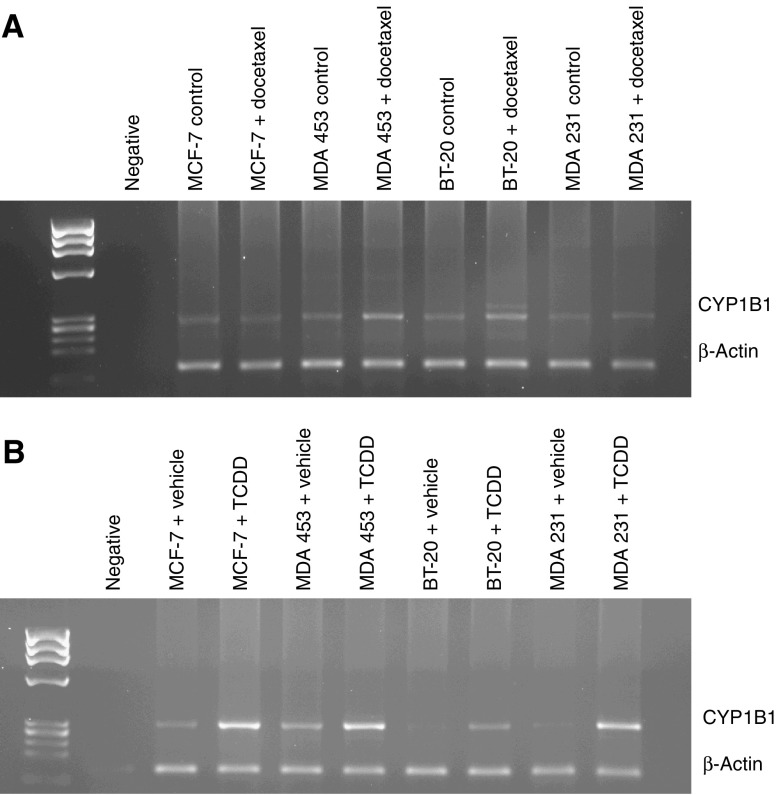
(**A**) Expression of CYP1B1 by RT–PCR in a panel of breast cell lines after docetaxel treatment. Cells were exposed to 80 ng ml^−1^ docetaxel or medium for 4 h before mRNA extraction. (**B**) Expression of CYP1B1 by RT–PCR in a panel of breast cell lines after TCDD treatment. Cells were treated with 3.22 ng ml^−1^ TCDD or vehicle (toluene) for 4 h before mRNA extraction.

Upregulation of CYP1B1 mRNA expression in MCF-7 cells was observed after long-term docetaxel treatment; however, it was not clear whether docetaxel *per se* was able to induce CYP1B1 mRNA expression or whether this effect was simply associated with the development of resistance. To clarify this, the effect of a short-term treatment with docetaxel on CYP1B1 mRNA expression was analysed in a panel of breast cell lines: MCF-7, MDA 453, BT-20 and MDA 231. CYP1B1 is commonly expressed in hormone-dependent tissue, such as prostate, breast and endometrium, and its expression can be affected by the ER status of cells. We therefore chose cell lines with different ER status: MCF-7 and MDA 453 cells are ER positive, while BT-20 and MDA 231 cells are ER negative. These cells were exposed to 80 ng ml^−1^ of docetaxel for 4 h, and expression of CYP1B1 was then measured by RT–PCR and compared to that of untreated cells. As can be seen in Figure 3A, a single 4-h pulse of docetaxel did increase expression of CYP1B1 mRNA in MDA 453 and BT-20 cells; a slight increase was also observed for MDA 231 cells. No apparent effect on expression was observed in MCF-7 cells after short-term docetaxel treatment. These results suggest that induction of CYP1B1 mRNA expression is not dependent on ER status.

To find out whether activation of the AhR can induce CYP1B1 expression in the panel of cell lines studied, cells were treated with TCDD, a well-known AhR agonist, for 4 h. Expression of CYP1B1 mRNA was then analysed and revealed upregulation of this enzyme in all cell lines tested in response to TCDD treatment (Figure 3B). This result indicates that TCDD is able to induce CYP1B1 mRNA expression via the AhR in the panel of breast cell lines, independently of ER status.

### CYP1B1 siRNA transfection in MCF-7 Txt cells

CYP1B1 is upregulated in MCF-7 Txt cells, which show an approximate three-fold increase in docetaxel resistance. To investigate whether CYP1B1 expression could influence docetaxel resistance in these cells, siRNA transfections were carried out to knock down the expression of this gene. Three predesigned siRNAs were used for CYP1B1; cells were transfected and then exposed to different concentrations of docetaxel.

**Figure 4:**
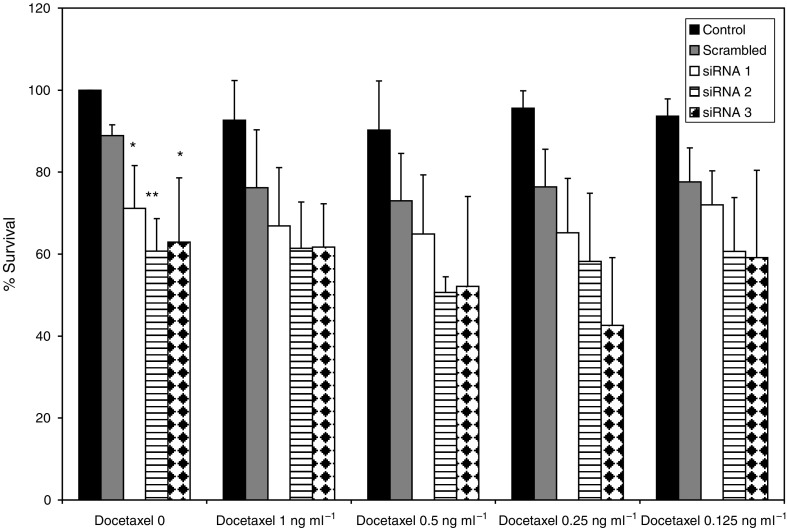
Effect of CYP1B1 siRNA transfection on docetaxel toxicity in MCF-7 Txt cells. Control cells received only fresh medium. Scrambled siRNA-transfected cells received a nonsense siRNA sequence. Results represent the average of four independent experiments. ^*^Indicates *P*<0.05. ^**^Indicates *P*<0.01.

As seen in Figure 4, transfection of CYP1B1 siRNAs 1, 2 and 3 did not result in appreciable enhancement of docetaxel toxicity. Remarkably, all three CYP1B1 siRNAs significantly decreased cell survival in the absence of drug (*P*<0.05), suggesting that the knockdown of this enzyme is detrimental to the viability of MCF-7 Txt cells.

### CYP1B1 metabolism and drug toxicity

**Figure 5:**
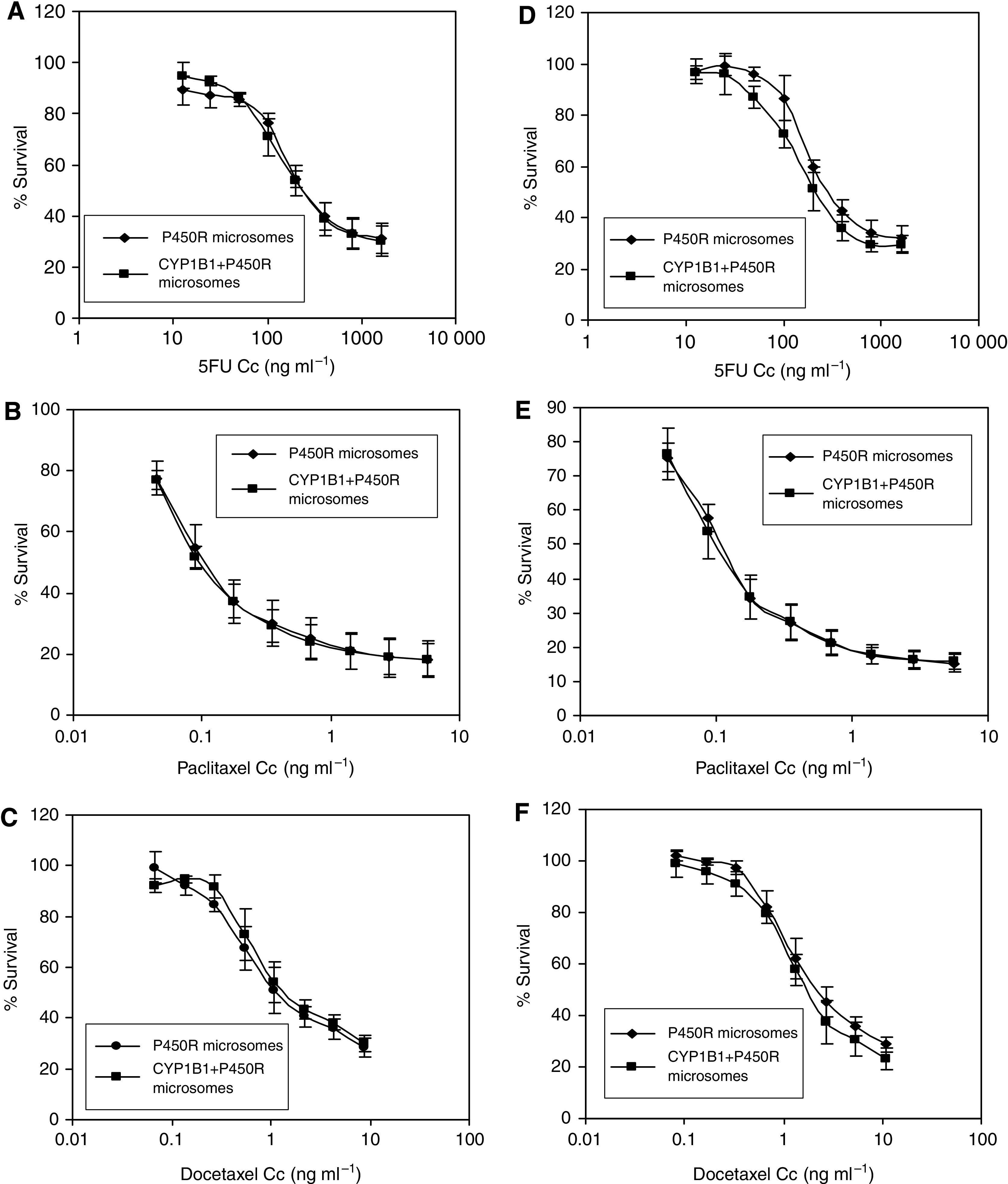
Drug toxicity in A549 cells as determined by the acid phosphatase assay. A known concentration of either 5-fluorouracil (**A** and **D**), paclitaxel (**B** and **E**) or docetaxel (**C** and **F**) was incubated with insect cell microsomes containing either CYP1B1 and P450R or P450R alone at 37°C for 30 min (**A**–**C**) or 1 h(**D**–**F**) prior to addition into the cells. The reaction mixture also contained 25 mM MgCl_2_ and 6.5 mM NADPH in potassium phosphate buffer (PBS, pH 7.4). The results represent the average of three independent experiments.

To study the degree to which CYP1B1 activity affects the toxicity of anticancer drugs, *in vitro* toxicity assays were performed on A549 cells; these cells display no measurable EROD activity (data not shown). A549 cells were exposed to different concentrations of docetaxel, paclitaxel and 5-fluorouracil, which had been preincubated with microsomes containing either human CYP1B1 and cytochrome *P*450 NADPH reductase (P450R) or P450R cDNA alone. Independent experiments were carried out, in which the drug was incubated with microsomes for either 30 min or 1 h. Figures 5A and B show that preincubation with CYP1B1 does not affect the toxicity of any of the drugs tested on A549 cells, at any of the two time points analysed.

## DISCUSSION

The development of chemotherapy resistance in breast cancer is considered to be multifactorial, involving several different mechanisms (O'Driscoll and Clynes, 2006). These include multidrug efflux pumps such as MDR1, MRP1 and BCRP, but also changes in expression of target proteins and growth factor receptors. Resistance is also developed against antihormonal treatment and aromatase inhibitors, highlighting the importance of hormones in tumour homeostasis (Ring and Dowsett, 2004; Dowsett *et al*, 2005; Weinberg *et al*, 2005).

Cytochrome *P*450 1B1 (CYP1B1) has been associated with the malignant phenotype after a number of studies found the expression of this P450 to be upregulated in malignant as compared to normal tissue. Further investigations revealed that CYP1B1 transfection increased docetaxel resistance in V79 cells (McFadyen *et al*, 2001). However, no CYP1B1-generated docetaxel metabolite has been detected to date, even after incubation of this drug with recombinant CYP1B1 in a number of different conditions (Bournique and Lemarie, 2002). The link between CYP1B1 expression and docetaxel resistance therefore remains unexplained.

It is a common feature of cytochromes *P*450 that the expression of a particular enzyme is induced by its substrate. To find out whether CYP1B1 expression could be induced by docetaxel, expression of this enzyme was analysed in MCF-7 cells and also in their resistant variants developed in our laboratory by pulse selection with cisplatin, adriamycin and docetaxel. These cells developed resistance to the drugs they were pulsed with: MCF-7 Txt cells showed an approximate three-fold increase in resistance to docetaxel as compared to parent MCF-7 cells. CYP1B1 mRNA levels were noticeably increased in MCF-7 Txt cells as compared to parent and adriamycin-selected cells. Ethoxyresorufin-*O*-deethylase activity levels, undetectable in MCF-7 parent cells, were low but readily measurable in MCF-7 Txt cells.

To find out whether pulse selection with docetaxel had a general effect on CYP1B1 expression, a panel of docetaxel-selected cell lines – colon carcinoma Caco2, pancreatic carcinoma BxPc-3 and squamous cell lung carcinoma SK-MES-1 – and their parental counterparts were subjected to RT–PCR analysis. CYP1B1 mRNA expression was undetectable in Caco2, BxPc-3 or SK-MES-1 parent or pulse-selected cells, suggesting that the effect of docetaxel on CYP1B1 expression is tissue-specific.

The effect of docetaxel on CYP1B1 mRNA expression was further analysed in a panel of breast cell lines, ER-positive MCF-7 and MDA 453 cells and ER-negative BT-20 and MDA 231 cells. Reverse transcription polymerase chain reaction results showed that a short docetaxel treatment induced a modest increase in the expression of CYP1B1 mRNA in MDA 453 and BT-20 cells, suggesting that this effect is not dependent on ER status; only a slight increase in CYP1B1 mRNA expression was observed in MDA 231 cells. Curiously, the effect was not observed in MCF-7 cells. It should be noted that changes in expression were evaluated in MCF-7 cells after a short-term docetaxel treatment, while MCF-7 Txt cells have been exposed to this drug on a regular basis and for a much longer period of time. These cells also display changes in their resistance profile, so in this case the induction of CYP1B1 mRNA expression could be associated with the observed increase in resistance.

CYP1B1 expression is regulated through the AhR pathway. To confirm the functionality of this pathway, the panel of breast cell lines was also exposed to the classic AhR agonist TCDD for 4 h. Induction of CYP1B1 mRNA was observed in all cell lines irrespective of ER status, suggesting that disruption of the AhR pathway is not a cause of the observed lack of acute CYP1B1 mRNA induction by short-term docetaxel treatment in MCF-7 cells.

Transfection of three different CYP1B1 siRNAs directed to three different sites in the CYP1B1 mRNA molecule resulted in decreased cell survival in basal conditions (i.e., in the absence of drug). This suggests that reduced CYP1B1 mRNA levels are detrimental to cells. The effect was not dramatic, but it was appreciable and indeed significant. In contrast, no major effect on docetaxel toxicity was detected in CYP1B1 siRNA-transfected MCF-7 Txt cells.

Our results with the siRNA experiments were quite unexpected. It is known that CYP1B1 generates toxic metabolites from oestrogen breakdown (Liehr, 2000); downregulation of CYP1B1 expression and consequent reduction in these toxic metabolites would then be expected to result in increased survival. However, we have found the opposite effect in MCF-7 Txt cells. It has been shown that 4-hydroxy oestrogen, the main CYP1B1 oestrogen metabolite, promotes MCF-7 cell proliferation (Schutze *et al*, 1993); a decrease in the levels of this metabolite following CYP1B1 downregulation could explain the reduction in cell number observed after CYP1B1 siRNA transfection. Although our results show that CYP1B1 expression levels can affect cell survival, the precise mechanism for this effect is not clear and further experiments need to be performed. To study the contribution of genotoxic oestrogen metabolites, we plan to carry out CYP1B1 siRNA transfections in an oestrogen-depleted environment using charcoal-stripped FCS and phenol red-free medium. It would also be worthy to further analyse the contribution of oestrogen to docetaxel-induced CYP1B1 expression, due to the presence of an oestrogen-responsive element in the gene promoter (Tsuchiya *et al*, 2004). Finally, it would be interesting to analyse the effect of docetaxel on CYP1B1 expression in other cell lines from hormone-dependent tissues, such as ovary and endometrium.

The microsomal fractions of insect cells expressing recombinant human cytochrome *P*450 cDNAs are a useful tool for the study of P450-mediated drug metabolism, since they display significant levels of enzymatic activity. Preincubation of 5-fluorouracil, paclitaxel or docetaxel with CYP1B1-containing microsomes did not affect the toxicity induced by these drugs in A549 cells, indicating that direct interaction of these chemotherapeutic drugs with CYP1B1 does not reduce their toxic effects.

Our results suggest that CYP1B1 does not mediate the direct inactivation of docetaxel and that other factors are responsible for the observed increase in docetaxel resistance in CYP1B1-transfected cells. It is possible that the increased levels of CYP1B1 reported in tumour as compared to normal tissue are simply due to the fact that the expression of this enzyme is associated with carcinogenesis and/or with the resistant phenotype, rather than being directly involved in drug resistance. However, knockdown of CYP1B1 expression appears to be detrimental to some cells; this suggests that CYP1B1 is not a simple resistance-associated marker but an enzyme with a role in promotion of survival. CYP1B1 does not appear to enhance docetaxel resistance by drug inactivation, so the effect is likely to be exerted through an indirect mechanism. It could be speculated that an endogenous metabolite of this enzyme is responsible for the promotion of cell survival, but further studies are needed to clarify this.

## Figures and Tables

**Table 1:** 5-Fluorouracil (5-FU), adriamycin, cisplatin and docetaxel IC_50_ values in MCF-7 cells and their pulse-selected variants as determined by the acid phosphatase assay

**IC_50_ values (ng ml^−1^)**	**MCF-7**	**MCF-7 CisPt**		**MCF-7 Txt**		**MCF-7 Adr**	
5-FU	336±87	234±15	**0.7**	270±27	**0.8**	579±100	**1.4**
Adriamycin	43.1±2.7	47.0±9.5	**1.1**	58.0±5.9	**1.3**	110±14	**2.6** ^*^
Cisplatin	580±120	1243±729	**2.1**	489±219	**0.8**	275±25	**0.5** ^*^
Docetaxel	1.1±0.2	2.1±0.3	**1.8**	3.1±0.7	**2.8** ^*^	10.5±3.6	**4.8**

Results are expressed as mean ±s.d. and represent the average of four independent experiments. Fold resistance of each variant is shown in bold and represents the IC_50_ value of the variants divided by the IC_50_ value of the parental cells for each particular drug tested. ^*^Indicates significance (*P*⩽0.05).

**Table 2:** EROD activity of MCF-7 cells

	**EROD activity (picomoles resorufin per mg total protein per min)**
MCF-7	ND
MCF-7 Txt	0.011±0.001

EROD=ethoxyresorufin-*O*-deethylase; ND=not detectable.

After addition of 7-ethoxy resorufin, cells were incubated at 37°C for 40 min. Samples were removed and spun down, and the amount of resorufin present in the supernatant determined by fluorescence (530 nm excitation; 590 nm emission). Results represent the average of five independent experiments. Activity is expressed as picomoles resorufin per mg of total protein per min.
